# Altered coronary artery calcium scores before bariatric surgery

**DOI:** 10.1186/2193-1801-3-199

**Published:** 2014-04-22

**Authors:** Patricia S Gadelha, Josemberg M Campos, Fernando Moraes, Mariana da F S Leão, Álvaro A B Ferraz

**Affiliations:** Division of Endocrinology, Hospital das Clínicas, Universidade Federal de Pernambuco, Av. Prof. Moraes Rego, 1235-Cidade Universitária, Recife, PE CEP: 50670-901 Brazil; Division of Surgery, Hospital das Clínicas, Universidade Federal de Pernambuco, Av. Prof. Moraes Rego, 1235-Cidade Universitária, Recife, PE CEP: 50670-901 Brazil; Division of Radiology, Real Hospital Português, Av. Agamenon Magalhães, Nº 4760, Recife, PE CEP: 52010-902 Brazil

**Keywords:** Coronary artery calcium score, Coronary artery disease, Obesity, Bariatric surgery, Cardiovascular risk factors

## Abstract

**Introduction:**

Obesity is an important cause of cardiovascular disease, especially coronary artery disease. Severely obese patients are particularly prone to this risk. The coronary artery calcium (CAC) score is a strong predictor of coronary heart disease and provides incremental information beyond traditional risk factors. We sought to determine the prevalence of abnormally high CAC scores in the preoperative setting among patients undergoing bariatric surgery and to establish risk predictors for higher scores.

**Methods:**

We performed an observational study of 202 patients free of known coronary artery disease who were referred for bariatric surgery. In each patient, the presence of CAC was detected with computed tomography, and coronary risk variables were either measured or determined via questionnaire.

**Results:**

CAC was found in 14.4% of the overall population (26% of male and 10.5% of female patients). Participants with altered CAC scores were older (mean age, 46.8 years). The variables positively associated with an altered CAC score were older age, male sex, type 2 diabetes, hypertension, and hypercholesterolemia. Multivariate-adjusted analysis showed that age (OR, 1.11; 95% CI, 1.06–1.17; p = 0.001), male sex (OR, 4.17; 95% CI, 1.52–11.47; p = 0.006), and hypercholesterolemia (OR, 6.21; 95% CI, 1.81–21.29; p = 0.004) were most closely related to the presence of CAC.

**Conclusion:**

Obese patients in the preoperative bariatric surgery setting have a high prevalence of abnormal CAC scores. Traditional risk factors play a important role in this scenario.

## Introduction

More than one-third (35.7%) of United States adults are obese (CDC 2013). Obesity carries a high risk of comorbidities, including type 2 diabetes, systemic arterial hypertension, and dyslipidemia (De Sa et al. 2011; Pajecki et al. 2007). These are traditional risk factors for cardiovascular diseases, particularly for coronary heart disease (CHD) (Kopelman 2000). Evidence-based guidelines recommend that physicians assess their patients’ baseline CHD risk and focus on primary preventative interventions (Expert Panel 2001).

Although coronary risk stratification is widely recommended, prediction models based on CHD risk factors are limited in their ability to discriminate individuals who will or will not develop CHD, leaving a large proportion of patients classifiable as having “intermediate” risk (Diverse Populations Collaborative Group 2002). This category includes individuals with one or more risk factors that exceed desirable levels, including obesity (Greenland et al. 2001).

Given this uncertainty, recent guidelines have highlighted the potential use of anatomically based CHD assessment to refine this risk prediction (Greenland et al. 2004, 2010). One such approach uses computed tomography (CT) to detect coronary artery calcium (CAC). The finding of CAC is related to an increased risk of incident CHD (Detrano et al. 2008) and is a proven objective, cost-effective, and independent prognostic tool in predicting this risk (Greenland et al. 2004; Taylor et al. 2005).

Obesity, especially visceral adiposity, has been associated with abnormally high CAC scores (Ho et al. 2009) and CAC progression (Kramer et al. 2009). However, little is known about CAC results in the specific population of severely obese individuals for whom bariatric surgery is indicated. Therefore, the aims of the present study were to assess the prevalence of abnormally high CAC scores in the preoperative bariatric surgery setting and establish risk predictors for higher scores.

## Methods

This study was carried out at the Universidade Federal de Pernambuco in Brazil and included all eligible patients who were referred for bariatric surgery from March 2012 to July 2013. A total of 202 patients met the following inclusion criteria: (i) body mass index (BMI) of ≥40 or ≥35 kg/m^2^ and one or more severe comorbidities according to the National Institutes of Health guidelines for bariatric surgery (Gastrointestinal surgery for severe obesity: National Institutes of Health Consensus Development Conference Statement 1992) and (ii) the absence of known coronary artery disease at the beginning of the study, including angina, myocardial infarction, and coronary revascularization. All patients gave their informed consent before participating in the present study. The institutional ethics committee (Ethic Committee of Health Sciences Center of Universidade Federal de Pernambuco) approved the study.

All study participants underwent questioning by a physician regarding the presence of any of the traditional risk factors, including a history of hypertension, type 2 diabetes mellitus, hypercholesterolemia, current medications, and current smoking. Fasting blood samples were collected to measure the levels of total cholesterol, low-density lipoprotein (LDL) cholesterol, high-density lipoprotein cholesterol, triglycerides, serum glucose, hemoglobin A1C and insulin. Anthropometric measurements including height and weight were also collected, and BMI was calculated as weight/height^2^ (kg/m^2^).

Hypertension was defined as the current use of antihypertensive medication or known and untreated hypertension. Diabetes was defined as a fasting blood glucose level of ≥126 mg/dL, a hemoglobin A1C level of ≥6.5%, or the use of antidiabetic medications. Hypercholesterolemia was defined as a total cholesterol level of >200 mg/dL or the current use of lipid-lowering therapy. Hypertriglyceridemia was defined as a triglyceride level of >150 mg/dL. Cigarette smoking was considered to be present if the patient was a smoker at the time of the survey.

**Figure 1:**
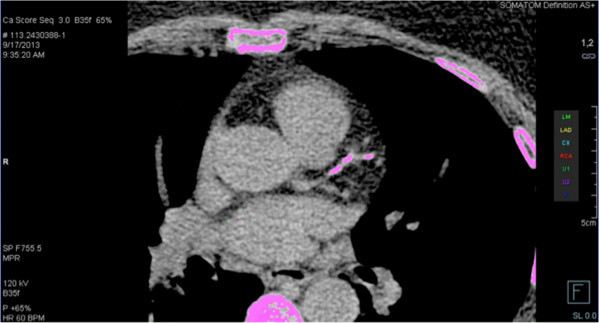
**Coronary artery calcification (CAC) of the left anterior descending coronary artery.** Workstation screen displaying a noncontrast enhanced axial image postprocessed with a calcium scoring preset: areas > 130 HU are coloured. A region of interest has been manually drawn, encircling areas at the main trunk and the distal segment of the left anterior descending coronary artery.

All patients underwent CAC scoring with a 128-row multidetector CT scanner (Siemens Somatom Definition AS, Siemens Medical Systems, Erlangen, Germany). Forty-eight contiguous CT slices were obtained at 3-mm intervals beginning 1 cm below the carina and progressing caudally to include the entire coronary tree. The image acquisition was triggered to 60% to 80% of the electrocardiographic R–R interval. Scans were interpreted by an experienced radiologist using the Agatston scoring method (Agatston et al. 1990). A focus of CAC was defined as the presence of three or more contiguous pixels with >130 Hounsfield units. The total CAC score was calculated as the sum of the individual scores of the three major epicardial coronary arteries. A scan was considered positive for CAC when the total CAC score was >0 (Figure 1).

Baseline characteristics in terms of the presence of CAC were computed as means or percents, and differences were tested using *t* tests or chi-square statistics, respectively. Multiple logistic regression after adjustment of univariate variables was conducted for age, sex, and hypercholesterolemia. Odds ratios (ORs) and 95% confidence intervals (CIs) were approximated. All statistical analyses were performed with a statistical software package (SPSS 18.0; SPSS Inc., Chicago, IL), and a p value of <0.05 was considered statistically significant.

## Results

Among the 202 total patients, 152 (75.2%) were female, and the mean age of the population was 36.3 years. The mean BMI was 40.5 kg/m^2^. Hypertension, type 2 diabetes mellitus, hypercholesterolemia, hypertriglyceridemia, and current smoking were present in 98 (48.5%), 40 (19.8%), 109 (54.0%), 89 (44.1%), and 13 (6.4%) patients, respectively (Table 1).

**Table 1:** **Demographic and biochemical characteristics of participants**

Variables	N = 202
Age (yrs, mean ± SD)	36.3 ± 10.3
Female sex, *n* (%)	152 (75.2%)
BMI (kg/m^2^, mean ± SD)	40.5 ± 4.3
Diabetes mellitus, *n* (%)	40 (19.8%)
Hypertension, *n* (%)	98 (48.5%)
Current smoker, *n* (%)	13 (6.4%)
Hypertriglyceridemia, *n* (%)	89 (44.1%)
Hypercholesterolemia, *n* (%)	109 (54.0%)

*BMI* body mass index, *SD* standard deviation.

CAC was present in 14.4% (n = 29) of the overall population (26% of male and 10.5% of female patients). The mean age of the patients with detectable CAC was 46.8 years. Of note, the presence of CAC did not lead to any clinical perioperative cardiovascular adverse outcome.

Univariate analyses were performed to identify factors that affect the presence of CAC. The presence of CAC was associated with male sex, age, diabetes, hypertension, and hypercholesterolemia (all p < 0.001) (Table 2).

**Table 2:** **Univariate analysis of variables influencing the presence of CAC**

Variables	CAC		***P*** value
	0 (n = 173)	>0 (n = 29)	
Male	37 (21.4%)	13 (44.8%)	0.007
Mean Age (SD)	34.3 (8.9)	46.8 (11.9)	< 0.001
Mean BMI (SD)	40.4 (4.4)	41.2 (3.8)	0.358
Diabetes	27 (15.6%)	13 (44.8%)	< 0.001
Hypertension	77 (44.5%)	21 (72.4%)	0.005
Current smoking	11 (6.4%)	2 (6.9%)	> 0.999
Hypertriglyceridemia	76 (43.9%)	13 (44.8%)	0.928
Hypercholesterolemia	84 (48.6%)	25 (86.2%)	< 0.001

*CAC* coronary artery calcification, *BMI* body mass index, *SD* standard deviation.

Multivariate analysis was then performed including variables that reached statistical significance in the univariate analyses. The presence of CAC was associated with male sex (OR, 4.17; 95% CI, 1.52–11.47; p = 0.006), older age (OR, 1.11; 95% CI, 1.06–1.17; p = 0.001), and hypercholesterolemia (OR, 6.21; 95% CI, 1.81–21.29; p = 0.004) (Table 3).

**Table 3:** **Multivariate analysis of presence of CAC**

Parameter	OR	95% CI	***P*** value
Age	1.11	1.06–1.17	0.001
Male gender	4.17	1.52–11.47	0.006
Hypercholesterolemia	6.21	1.81–21.29	0.004

*CAC* coronary artery calcification, *OR* odds ratio, *CI* confidence interval.

## Discussion

CAC represents a measure of the overall cardiac plaque burden (Budoff et al. 2006) and is a proven independent predictor of cardiovascular events (Detrano et al. 2008; Taylor et al. 2005). Previous studies have demonstrated an association between obesity and the presence of CAC. In the Multi-Ethnic Study of Atherosclerosis (MESA), Burke et al. (2008) demonstrated that obesity was associated with a 1.2-fold greater prevalence of CAC. However, there was a lack of data regarding the CAC score in a specific population of preoperative severely obese patients undergoing bariatric surgery.

The present study showed a prevalence of altered CAC scores in our population of 14.4%, 26% of males and 10.5% of females. Taylor et al. (2005) reported altered CAC scores in 22.4% of men and 7.9% of women in the Prospective Army Coronary Calcium Project (PACC), which analyzed 2000 participants with a mean age of 43 years. Tota-Maharaj et al. (2012) reported a prevalence of high CAC scores in 5% of their study subgroup, which had a mean age of 40 years. Considering that our study population had a lower mean age than that of other studies (36.3 years) and we still found a higher prevalence of abnormal CAC scores than that found in studies of nonobese subjects, we can assume that severe obesity is an important contributor to vascular calcification.

Our results also showed that age and male sex are associated with the presence of CAC. Previous studies have reported that older age is a main risk factor of higher CAC scores (Tota-Maharaj et al. 2012; McClelland et al. 2006; Budoff et al. 2007). McClelland et al. (2006) reported that the relationship between the probability of any detectable calcium and age is linear. The prevalence of CAC was higher in male patients, with 4-fold greater risk of a CAC score of >0. This finding is also compatible with those of previous studies (Taylor et al. 2005; McClelland et al. 2006; Loria et al. 2007).

Type 2 diabetes mellitus and hypertension were associated with high CAC scores in our study. Both are known traditional risk factors and have been associated with the presence of CAC in previous reports (Budoff et al. 2007; Loria et al. 2007; Hoff et al. 2003). Hypercholesterolemia was the strongest single variable associated with high CAC scores. The presence of hypercholesterolemia was associated with a 6-fold risk of having CAC. This finding can be understood in view of the fact that obese patients have an increased concentration of small, dense LDL particles. These LDL particles are more prone to oxidation and can move through endothelial fenestrations and enter the subendothelial space, where inflammation and transformation into plaque can occur (van Gaal et al. 2006).

The lack of a significant difference in CAC scores between smokers and nonsmokers, a finding that is in disagreement with previous studies (Loria et al. 2007; Budoff et al. 2009), could be due to the low prevalence of smoking in our population. A larger sample size would be required to identify a statistically significant difference.

The results of our study should be interpreted in the context of some limitations. First, it was a cross-sectional study and thus did not provide long-term outcomes data for the included patients. Second, some variables were measured while others were self-reported, and the potential of under-reporting might be considered. Finally, the patients were self-referred subjects; this may have caused bias because the study population may not represent the whole population of severely obese patients, who, in many developed countries, do not have access to medical treatment for obesity and its consequences.

## Conclusion

This study provides valuable information regarding CAC scores in severely obese patients, which may help physicians to encourage patients to achieve changes in modifiable risk factors and thus prevent, or at least delay, coronary artery calcification.

## Abbreviations

BMI: Body mass index
CAC: Coronary artery calcium
CI: Confidence intervals
CT: Coronary tomography
CHD: Coronary heart disease
LDL: Low-density lipoprotein
MESA: Multi-Ethnic Study of Atherosclerosis
OR: Odds ratio
PACC: Prospective Army Coronary Calcium Project.
